# Anti-contactin-associated protein-like 2 antibody-associated encephalitis in children: A case report and literature review

**DOI:** 10.3389/fped.2022.1004210

**Published:** 2022-10-20

**Authors:** Qingyang Dou, Renke Li, Xiaomei Shu

**Affiliations:** ^1^ Department of Pediatrics, Affiliated Hospital of Zunyi Medical University, Zunyi, China; ^2^ Department of Pediatrics, Guizhou Provincial People's Hospital, Guiyang, China

**Keywords:** autoimmune encephalitis, caspr2, antibody, prognosis, children

## Abstract

**Background:**

Anti-Contactin-associated protein-like 2 (CASPR2) antibody-associated encephalitis is a rare group of autoimmune diseases that causes extensive damage to the central and/or peripheral nervous system.

**Case presentation:**

Here, we reported a case of anti-CASPR2 antibody-associated encephalitis in a 12-year-old male patient with symptoms of headache, consciousness disturbance, mental abnormalities, urinary incontinence, fasciculations in the extremity muscles, and involuntary movements. The testing for autoimmune encephalitis-associated antibodies showed that CASPR2-associated antibodies were positive, and electroencephalography showed diffuse slow waves. No tumor was found after screening for malignancies. The child's status significantly improved after receiving immunotherapy with intravenous methylprednisolone and immunoglobulin.

**Conclusions:**

Anti-CASPR2 antibody-associated encephalitis has been rarely reported in children. It has a complex clinical presentation and a low incidence of tumor. Most pediatric patients have a favorable prognosis and relapse is uncommon.

## Introduction

Contactin-associated protein-like 2 (CASPR2) is a cell adhesion protein of the neurexin IV superfamily that is widely distributed in the neuronal membranes of the central nervous system (CNS) and peripheral nervous system (PNS) (1). It facilitates proper localization and aggregation of the voltage-gated potassium channel (VGKC) complexes in myelinated axons, stabilizes conduction at nodes of Ranvier, and maintains resting potential (2). Antibodies against CASPR2 bind to multiple antigenic epitopes on the CASPR2 protein and cause pathogenicity by altering the CASPR2-associated protein-protein interactions (1). CASPR2-associated antibodies impair CNS and PNS with various clinical syndromes, such as limbic encephalitis (LE), Morvan syndrome, peripheral nerve hyperexcitability (PNH), cerebellar syndrome, neuropathic pain, and autonomic dysfunction (3, 4).

Anti-CASPR2 antibody-associated encephalitis most commonly affects senior male patients (5), However, there are only a few reports concerning pediatric cases. Hence, in this study, we report a case of pediatric autoimmune encephalitis with positive CASPR2-associated antibodies. Additionally, we comprehensively review the literature to summarize the clinical characteristics, auxiliary examinations, treatment response, and prognosis of the disease to provide a valuable reference for early diagnosis and treatment of anti-CASPR2 antibody-associated encephalitis in children.

## Materials and methods

### Case report

This study reports a pediatric case of anti-CASPR2 antibody-associated encephalitis from the Department of Pediatrics, First Affiliated Hospital of Zunyi Medical University. We acquired the patient's basic information, clinical symptoms, and test results from the e-medical database. The patient was followed up either over the phone or in an outpatient setting. The child's parents provided written informed consent, and this study was approved by the Ethics Committee of Affiliated Hospital of Zunyi Medical University.

### Literature review

We analyzed the characteristics of pediatric patients with anti-CASPR2 antibody-associated encephalitis by incorporating other cases from the literature. For this, we searched the following terms on PubMed, Web of Science, and Embase (up to July 2022): “encephalitis,” “CASPR2,” and “Contactin-associated protein-like 2”. This study included publications with a definite diagnosis of autoimmune encephalitis and patients <18 years, whereas excluded those written in non-English or with inadequate clinical information.

## Results

### Case presentation

A 12-year-old boy was admitted to the Affiliated Hospital of Zunyi Medical University with headache, disturbance of consciousness, mental abnormalities, and urinary incontinence for 1 day, and without any history of diseases. On admission, the boy presented symptoms of fasciculations in the extremity muscles and meningeal irritation. The physical examination results were as follows: the temperature was 37 °C, pulse was 90 beats/minute, respiration was 22 breaths/minute, and blood pressure was 110/72 mmHg.

**Figure 1:**
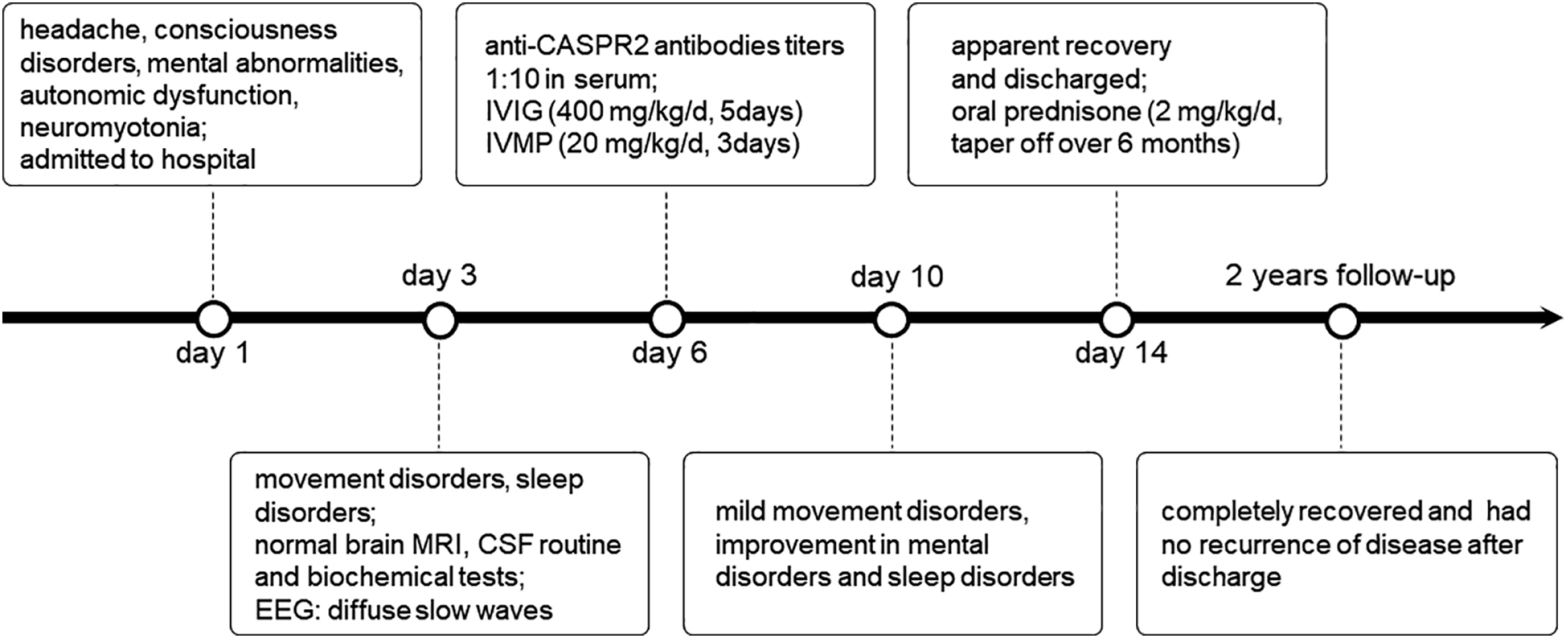
Timeline of disease onset and course.

On the first day of admission, the child exhibited insomnia, orofacial spasms, and involuntary movements of the upper and lower limbs intermittently (timeline in Figure 1). His brain magnetic resonance imaging (MRI) was negative. The lumbar puncture pressure was 120 mm H20, and the cerebrospinal fluid (CSF) routine and biochemical tests were normal. Electroencephalography (EEG) showed diffuse slow waves on the third day of admission. On assessing the serum tumor markers and performing a CT of the chest and abdomen, we found no tumor. The CSF and serum samples of the child were sent to the Wuhan Kindstar Diagnostics Clinical Lab for commercial cell-based assays and indirect immunofluorescence tests to detect autoimmune encephalitis-associated antibodies, such as NMDAR, AMPAR, CASPR2, LGI1, GABAB, GlyR, Hu, Yo, Ri, CV2, Ma2, and amphiphysin. The anti-CASPR2 antibody was detected in the serum, but not in the CSF. We applied the ELISA method to detect the corresponding virus-specific antibodies in the CSF, including EV, HSV, EBV, ADV, and FLuV; however, there were no positive findings.

After a comprehensive judgment of clinical symptoms, EEG, and antibody test results, the child was diagnosed with anti-CASPR2 antibody-associated encephalitis. The child was administered intravenous immunoglobulin (IVIG, 400 mg/kg/d, 5 days) and intravenous methylprednisolone (IVMP, 20 mg/kg/d, 3 days). At the end of the IVMP period, the medication was altered to oral prednisone (2 mg/kg/d). The dosage was gradually tapered after 8 weeks for a total course of 6 months. The child's symptoms significantly improved after rapid immunotherapy, and he was discharged from the hospital with an apparent recovery. The child was followed up for 2 years and he completely recovered without recurrence. During the entire course of the disease, the child did not present the core symptoms exhibited by adult patients with anti-CASPR2 antibody-associated encephalitis, such as neuropathic pain or weight loss.

### Literature review

We identified nine children with anti-CASPR2 antibody-associated encephalitis from five published reports (6–11) (Table 1). Together with our case, the 10 pediatric cases are listed in Table 1. We considered the cases in publications by Kim et al. (6) and Sunwoo et al. (7) as the same case based on the similarities in basic information, laboratory findings, and prognosis of the cases. As a result, we consolidated the case information from these publications.

**Table 1:** Clinical characteristics of anti-CASPR2 antibody-associated encephalitis in children.

Reference	Sunwoo et al. [7] Kim et al. [6]	Zhang et al. [8]	Nagarajan et al. [9]	Tan et al. [10]	Kang et al. [11]	Our case
Number of cases	1	1	1	6	5	1
Age/range, Y	8	5	1.6	1.8–14	2.1–9.8	12
Sex	F	M	F	1F/5M	1F/4M	M
Clinical characteristics						
Prodromal symptoms	YES	YES	NO	3	1	YES
Psychiatric symptoms	YES	NO	YES	6	5	YES
Seizures	YES	YES	NO	2	3	NO
Speech disorders	NO	NO	YES	1	2	NO
Movement disorders	NO	NO	NO	6	1	YES
Altered consciousness	NO	NO	NO	3	3	YES
Autonomic dysfunction	NO	NO	NO	1	3	YES
Sleep disorders	NO	NO	YES	3	4	YES
Cerebellar ataxia	NO	NO	NO	NO	NO	NO
PNH	NO	NO	NO	NO	NA	YES
neuropathic pain	NO	NO	NO	NA	NA	NO
weight loss	NO	NO	NO	NA	NA	NO
CSF WBC >5/ml	YES	NO	NO	2	4	NO
Elevated CSF protein	NO	NO	NO	NO	1	NO
CASPR2-Ab	serum	serum	serum	6 serums	3 serum; 2 CSF and serum	serum
MRI	leptomeningeal enhancement	NO	NO	1 demyelination of the bilateral frontal lobes	2	NO
EEG	slow waves	slow waves	slow waves	5 slow waves, included 1 accompanied by epileptiform discharge	5 slow waves, included 2 accompanied by epileptiform discharge	slow waves
Tumor	NO	NO	NO	NO	NO	NO
Therapy	IVMP and IVIg	NO	IVMP and IVIg	6 IVMP and IVIg; 1 CPA; 1 RTX and MMF	5 IVMP and IVIg; 1 RTX	IVMP and IVIg
Outcome	partial recovery	Complete recovery	Complete recovery	5 Complete recovery; 1 partial recovery;	4 Complete recovery; 1 partial recovery;	Complete recovery
Follow-up, Mo	18	24	12	7–24	at least 6	24
Relapse	NO	NO	NO	NO	NO	NO

Ab, antibodies; CASPR2, The Contactin-associated protein-like 2; CPA, cyclophosphamide; CSF, cerebrospinal fluid; EEG, electroencephalography; F, female; IVMP, intravenous methylprednisolone; IVIg, intravenous immunoglobulin; M, male; MMF, mycophenolate mofetil; Mo, months; MRI, magnetic resonance imaging; PNH, peripheral nervous hyperexcitability; RTX, rituximab; WBC, white blood cells; Y, years.

### Demographic and clinical characteristics

Among the 15 pediatric cases, eleven (73%) were males, and the age of onset ranged from 1.6–14 years. Seven cases (47%) showed prodromal symptoms, including fever in two cases, headache in three cases, and fever accompanied by headache in two cases. The common clinical manifestations included psychiatric symptoms in fourteen cases (93%), sleep disorders in nine cases (60%), movement disorders in eight cases (53%), seizures in seven cases (47%), altered consciousness in seven cases (47%), speech disorders in four cases (27%), and autonomic dysfunction in five cases (33%). Acquired neuromyotonia was present only in the child we reported.

### Auxiliary examinations

The autoimmune encephalitis-related antibody tests were performed in all children, and CASPR2-associated antibodies were detected in all serum samples, while CSF samples were negative. The CSF examination showed leukocytosis (>5/mm3) in seven children (47%) and elevated protein in one case (7%). The brain MRI examination was abnormal in four cases (27%): two in frontal lobe, two in thalamus, one in parietal lobe, one in temporal lobe, one in occipital lobe, one in insular lobe, and one diffuse leptomeningeal enhancement. The EEG was abnormal in fourteen cases (93%): all cases showed slow waves and three were accompanied by epileptiform discharges. A chest or abdomen CT scan was performed on all children to screen for potential tumors, but no abnormalities were found.

### Treatment and prognosis

The treatment strategy followed the first-line immunotherapy protocol (corticosteroid, immunoglobulin, and plasma exchange). Except for one case without immunotherapy due to parental refusal, fourteen cases received a combination of IVMP and IVIG. Three cases used second-line immunotherapy with rituximab, mycophenolate mofetil, or cyclophosphamide. Antiepileptic medications were used in four cases, risperidone in three cases, and anti-tuberculosis treatment for obsolete pulmonary tuberculosis was used in one case.

All children were tracked for at least 6 months, with a maximum follow-up of 24 months. All children had a good prognosis, with complete recovery in twelve children and partial recovery in three children. During the follow-up period, no child experienced a relapse.

## Discussion

Anti-CASPR2 antibody-associated encephalitis mostly appears in adult patients, especially in the elderly. Here, we describe a rare pediatric case which has rarely been reported in the literature. The case was identified as anti-CASPR2 antibody-associated encephalitis based on the clinical presentation and detection of autoimmune encephalitis-related antibodies. A retrospective study of 103 pediatric autoimmune encephalitis cases showed that anti-CASPR2 antibodies were detected in serum samples from one child (8).

Autoantibody-mediated immunological diseases are more frequently observed in women. However, anti-CASPR2 antibody-associated encephalitis in males accounted for almost 90% of adult patients (3, 5). In line with other studies, this study included 11 male children (73%). The reason for this gender predominance is not clear; however, it could be related to the existence of CASPR2 mRNA in male prostates (12).

The anti-CASPR2 antibody-associated encephalitis has an acute, subacute, or chronic period of onset, with 30% of patients having >12 months. This disease exhibits an extensive clinical spectrum, with seven core symptoms in adult patients as follows: limbic system symptoms (cognitive impairment, seizures, and psychiatric symptoms), cerebellar ataxia, PNH (fasciculations and cramps), autonomic symptoms, sleep disturbances, neuropathic pain, and weight loss. 77% of adult patients presented ≥3 of these core symptoms, and 61% ≥ 4. Joubert et al. (5) observed that the characteristic symptoms of anti-CASPR2 antibody-associated encephalitis were prominent limbic system symptoms and temporal lobe epilepsy. A recent multicenter study on 164 adult patients showed that movement disorders and ataxia are prevalent in CASPR2 autoimmunity (13).

The clinical manifestations of anti-CASPR2 antibody-associated encephalitis are diverse and complex in children, including 14 cases of psychiatric symptoms, nine cases of sleep disorders, eight cases of movement disorders, seven cases of seizures, seven cases of altered consciousness, five cases of autonomic dysfunction. However, no reports have explicitly described the presence of cerebellar ataxia, neuropathic pain, and weight loss in pediatric cases. This suggests that the variety of clinical presentations may be lesser in pediatric patients than in adult patients. Only one case presented with PNH manifesting as acquired neuromyotonia. PNH is a specialized sign of anti-CASPR2 antibody-associated encephalitis, including fasciculations, cramps, and neuropathic pain. CASPR2-associated antibodies are pathogenic in patients with autoimmune neuromyotonia (14), and they may disrupt the CASPR2/TAG-1 interaction and shaker-type voltage-gated potassium channels (Kv1) expression, resulting in enhanced neuronal excitability (15).

The co-occurrence of the PNH and CNS symptoms is referred to as Morvan syndrome. The French physician Morvan first described the Morvan syndrome in 1890, which was characterized by PNH, autonomic dysfunction, and encephalopathy with significant insomnia. It was the most prominent clinical spectrum of pediatric neurological diseases with CASPR2 and leucine-rich glioma-inactivated protein 1 (LGI1) double-positive antibodies (16). Among the cases included, only the child that we reported was diagnosed with Morvan syndrome. This classical phenotype of anti-CASPR2 antibody-associated autoimmune diseases appears to be rarely observed in children. However, it cannot be excluded that this is related to the young age of some children in our study, who were incapable or had trouble identifying the symptoms of PNH.

The routine biochemical testing of CSF was not significantly distinctive, and most CASPR2-associated antibody-positive patients were normal (1, 17). Seven children showed CSF leukocytosis, and one child showed an increased level of protein, consistent with previous findings. Joubert et al. (5) showed the presence of CASPR2-associated antibodies in the CSF of aged patients with anti-CASPR2 antibody-associated autoimmune encephalitis only and serum of patients with neuromyotonia or Morvan syndrome. In contrast, 13 cases of this study detected CASPR2-associated antibodies in the serum and only two cases in the CSF and serum. This indicates that the antibody-negative testing in the CSF fails to exclude the diagnosis of anti-CASPR2 antibody-associated encephalitis in children, and both serum and CSF samples should be delivered if possible. Anti-CASPR2 antibodies can be identified in the serum of about 1% of healthy individuals and patients with various neuropsychiatric disorders (18). Therefore, clinical symptoms, imaging, additional laboratory tests, and elimination of other diseases are required in combination to diagnose this disease.

The brain MRI findings were abnormal in 53% of anti-CASPR2 antibody-positive adult patients, with unilateral or bilateral hyperintense T2 weighted image in the medial temporal lobes, hippocampal shrinkage, mesial temporal sclerosis, and hippocampal sclerosis (4). According to Bien et al. (19), MRI is a significant predictor of anti-CASPR2 antibody-associated encephalitis, and abnormal MRI findings can increase its diagnostic accuracy. In our review, four cases had abnormal brain MRI, including two in frontal lobe, two in thalamus, one in parietal lobe, one in temporal lobe, one in occipital lobe, one in insular lobe, and one in diffuse leptomeningeal enhancement. However, a more extensive sample investigation is needed to establish the abnormal rate and predictive value of brain MRI in pediatric patients with anti-CASPR2 antibody-associated encephalitis.

Most CASPR2-associated antibody-positive adult patients had normal EEG, with only 33% showing epileptic discharges and focal or diffuse slow waves (4). EEG abnormalities such as localized or diffuse slow waves were present in 14 of our cases (93%), three of which were combined with epileptic discharges. Thus, pediatric patients exhibited a higher rate of abnormal EEG than adult patients (90% vs. 33%), and the composition of abnormal forms was different from those in adults. This suggests a favorable diagnostic value of EEG for diagnosing anti-CASPR2 antibody-associated encephalitis in children.

It is essential to screen patients with anti-CASPR2 antibody-associated encephalitis for malignancies. In all, 21.8% of the anti-CASPR2 antibody-positive adult patients were screened positive for tumors. The most prevalent was thymoma, while there were a few cases of prostate cancer, melanoma, lung adenocarcinoma, endometrial carcinoma, and pancreatic adenocarcinoma (4, 20). In this study, tumor was not detected in any pediatric case. However, additional research is needed to confirm whether anti-CASPR2 antibody-associated encephalitis in children is linked to tumors.

Owing to the rarity of anti-CASPR2 antibody-associated encephalitis, it lacks a standardized treatment protocol. The primary treatments include immunotherapy, symptomatic therapy, and supportive treatment, and patients with a tumor should be treated aggressively. In this study, 14 children were treated with a combination of IVMP and IVIG. Of these, one had a poor outcome, probably due to a lack of initial IVMP, but symptoms showed significant improvement upon IVMP addition. Three children received second-line immunotherapy with rituximab, mycophenolate, or cyclophosphamide.

A study of anti-CASPR2 antibody-positive adult patients with follow-up (median time of 36 months) showed that 73% had a good prognosis (modified Rankin score ≤2), with a death rate of 3% after one year and 10% after two years. Relapse occurred in 25% of the patients, who presented comparable or different core symptoms from the initial period, and some patients could relapse after a 6-year interval. In half of the relapse cases, a definitive diagnosis was not made during the first period of the disease (3). A few adult patients with anti-CASPR2 antibody-associated encephalitis can have tumors during the relapse (21). All children included in this study had a good prognosis during the follow-up (at least 6 months), with 12 children having complete recovery, three children having partial recovery, and no child relapsing. Anti-CASPR2 antibody-associated encephalitis in children may have a better prognosis and a lower relapse rate than in adults. However, future studies with large samples are required to better investigate the issue.

## Conclusion

Here, we described a male child with anti-CASPR2 antibody-associated encephalitis and summarized the clinical features of pediatric cases with anti-CASPR2 antibody-associated encephalitis for the first time through a systematic literature review. Anti-CASPR2 antibody-associated encephalitis in children manifests as psychiatric symptoms, movement disorders, sleep disturbances, seizures, and altered consciousness. Tumor is infrequently found in children with this disease. The antibody-positive rate is higher in serum than in CSF. Most EEG examinations are abnormal, with focal or diffuse slow waves, but abnormal brain MRI is uncommon. Most pediatric cases have a good prognosis and less recurrence after immunotherapy. Therefore, early identification and treatment of this disease are essential. However, this study has insufficiencies, such as a small number of cases and the lack of electromyography. These can be addressed by increasing the sample size, enhancing the case data, and further intensive observation and study.

## Data Availability

The original contributions presented in the study are included in the article/Supplementary Material, further inquiries can be directed to the corresponding author/s.
